# Association between Complement C3 and Prevalence of Fatty Liver Disease in an Adult Population: A Cross-Sectional Study from the Tianjin Chronic Low-Grade Systemic Inflammation and Health (TCLSIHealth) Cohort Study

**DOI:** 10.1371/journal.pone.0122026

**Published:** 2015-04-09

**Authors:** Qiyu Jia, Chunlei Li, Yang Xia, Qing Zhang, Hongmei Wu, Huanmin Du, Li Liu, Chongjin Wang, Hongbin Shi, Xiaoyan Guo, Xing Liu, Shaomei Sun, Xing Wang, Ming Zhou, Honglin Zhao, Kun Song, Yuntang Wu, Kaijun Niu

**Affiliations:** 1 Health Management Centre, Tianjin Medical University General Hospital, Tianjin, China; 2 Nutritional Epidemiology Institute and School of Public Health, Tianjin Medical University, Tianjin, China; National Taiwan University College of Medicine, TAIWAN

## Abstract

Activation of the innate immune system plays a key role in the development of fatty liver disease (FLD). The complement system is a major humoral component of the innate immune response and complement C3 plays a central role, implying that C3 may be a powerful predictor or therapeutic target for FLD. However, few studies have assessed the association between C3 and FLD in a large population. Here we use a cross-sectional study to investigate the link between serum C3 levels and FLD. Participants were recruited from Tianjin Medical University’s General Hospital-Health Management Centre. Serum C3 was measured using immunoturbidimetry method and FLD was diagnosed by liver ultrasonography. Multiple logistic regression analysis was used to examine the association between quartiles of C3 and FLD prevalence. The overall prevalence of nonalcoholic fatty liver disease (NAFLD) and alcoholic fatty liver disease (AFLD) were 37.3% and 10.1%, respectively. After adjusting for covariates, the odds ratio of having NAFLD or AFLD (only in males) in the fourth quartile of C3 compared with the first quartile was 4.13 times greater (95% confidence interval, 2.97-5.77) (trend *P* values < 0.0001) and 2.09 times greater (95% confidence interval, 1.08-4.18) (trend *P* values = 0.02). This is the first study to demonstrate that serum C3 levels are independently associated with a higher prevalence of NAFLD and AFLD (only in males) in an adult population. Further studies are needed to establish a causal link and determine the precise role of C3 in FLD.

## Introduction

Fatty liver diseases (FLD) is the most common liver dysfunction worldwide and can be categorized as nonalcoholic FLD (NAFLD) or alcoholic FLD (AFLD) according to etiology [1, 2]. Fatty liver diseases can lead to a spectrum of ranging from simple hepatic steatosis, steatohepatitis, liver fibrosis, cirrhosis and even hepatocellular carcinoma [3]. Risk of development of FLD is elevated by obesity, type 2 diabetes, dyslipidemia, hypertension and metabolic syndrome and other conditions; thus, a large proportion of Chinese people are at high risk of developing FLD [4]. FLD is emerging as a leading cause of chronic liver disease and increasing risk of overall, as well as cardiovascular and liver-related mortality [5–7]. The prevalence of FLD in China has approximately doubled over the past two decades, and approximately 27% urban population have been diagnosed with FLD [8].

Emerging evidence suggests that activation of the innate immune system contributes to FLD pathogenesis [9]. Complement plays a major role in human innate immunity, and complement proteins are produced mainly by the liver [10]. Complement C3, a central molecule of the complement system, has multiple roles in the liver, including pro-inflammatory and regenerative functions, and responding to exposure of toxins such as alcohol. Animal studies have suggested that C3 contributes to accumulation of triglycerides in the liver, while C5 appears to be involved in inflammation and injury to hepatocytes [11]. In humans, complement has been both directly and indirectly linked to liver damage. The results of one such study using liver biopsy strongly indicate that complement is altered in liver damage [12]. Another case-control study mentioned that C3 serum levels were higher in NAFLD patients [13] and one cross-sectional study concluded that C3a levels paralleled the degree of liver injury [14]. One study found that FLD and concomitant liver dysfunction (represented by alanine aminotransferase (ALT) levels), led to enhanced production of complement C3 [15]. Together, these results suggest that complement C3 may be a useful predictor or therapeutic target for the treatment or prevention of FLD. However, few studies have shown a direct correction between C3 levels and FLD in an adult population.

Therefore, we designed a cross-sectional study to investigate the association between serum C3 concentration and FLD in a large Chinese adult population.

## Methods

### Study population

The Tianjin Chronic Low-grade Systemic Inflammation and Health (TCLSIH or TCLSIHealth) Cohort Study is a prospective dynamic cohort study, the detail of which has been published elsewhere [16, 17]. Participants were recruited during annual health examinations at the Tianjin Medical University General Hospital-Health Management Center, the largest and most comprehensive physical examination center in Tianjin.

This cross-sectional study used baseline data from the TCLSIHealth. During the research period there were 2,999 participants who had received health examinations that included both serum C3 and abdominal ultrasound detection. We excluded participants for whom BMI was not available (n = 7), or for whom blood analysis was missing (n = 66). No participants were found to have liver disease (chronic hepatitis B or C, operation on liver, autoimmune liver diseases or liver cancer). The final cross-sectional study population comprised 2,926 participants (no FLD, n = 1,713; NAFLD, n = 1,020; AFLD, 186 in males and 7 in females). For analysis of participants with NAFLD, we excluded participants diagnosed with AFLD (n = 193). When assessing relationship between AFLD and serum C3, we excluded participants who had NAFLD (n = 1,020). Blood sample drawn from each subject were routinely 12 ml of whole blood for 2 ml of plasma and 10 ml of serum. This study was approved by the Institutional Review Board of the Tianjin Medical University and each participant had written informed consent before the study.

### Liver ultrasonography

Fatty liver was diagnosed by real-time ultrasonography using standardized criteria performed by experienced technicians [18]. Positive abdominal ultrasound images contained: diffusely increased liver near field ultrasound echo (‘bright liver’) and increased liver echotexture when compared to the kidneys; vascular blurring and the gradual attenuation of far field ultrasound echo. Diagnosis of FLD required at least two of the abnormal findings listed above [19]. Participants with sonographic fatty liver and a self-reported weekly alcohol intake of <140 g and <70 g for males and females, respectively, were classified as having NAFLD. Alcohol consumption >140 g and 70g for males and females, respectively, with sonographic fatty liver were considered to have AFLD.

### Assay of C3

Serum C3 concentration was measured using an immunonephelometric assay performed with an automated IMMAGE 800 immunochemistry system (Beckman Coulter, Brea, CA, USA), and expressed as g/L. The detection limit of the assay was 0.058 g/L, the measurement range was 0.058–126 g/L, and the intra- and inter-assay coefficients of variation (CV) were less than 4–6%. The manufacturer indicates that a C3 serum level of 0.79–1.52 g/L is typical of healthy adults.

### Assessment of other variables

Blood samples for analysis of fasting blood sugar (FBS) and lipids were collected in siliconized vacuum plastic tubes. FBS was measured using the glucose oxidase method, total cholesterol (TC) and triglycerides (TG) were measured by enzymatic methods, low density lipoprotein (LDL) cholesterol was measured by the polyvinyl sulfuric acid precipitation method, high-density lipoprotein (HDL) cholesterol was measured by chemical precipitation, ALT was measured by IFCC method, aspartate aminotransferase (AST) was measured by colorimetry and gamma-glutamyl transpeptidase (GGT) was measured by enzymic colorimetry using reagents from Roche Diagnostics on an automatic biochemistry analyzer (Roche Cobas 8000 modular analyzer, Mannheim, Germany).

Blood pressure (BP) was recorded as the mean of two measurements taken from the upper left arm at the brachial artery using an automatic device (Andon, Tianjin, China) after 5 minutes of rest in a seated position. Waist circumference was measured at the umbilical level with participants standing and breathing normally. Anthropometric variables (height and body weight) were recorded using a standard protocol. Body mass index (BMI) was calculated as weight in kilograms (kg) divided by height in squared meters (m^2^). Sociodemographic variables, including gender and age, were also assessed. Detailed information about personal and family history of physical illness, current medications (“yes” or “no”), and smoking behavior (“yes” or “no”) was provided by each participant. Alcohol consumption was estimated by a questionnaire survey, and expressed in grams per week.

### Statistical analysis

All statistical analyses were performed using the Statistical Analysis System 9.3 edition for Microsoft Windows (SAS Institute Inc., Cary, NC, USA). Descriptive data are presented as the mean (95% confidence interval) for adjusted continuous variables, and as percentages for categorical variables. Because the distribution of all continuous variables was non-normal, the natural logarithm was applied to normalize the data before analysis of variance (ANOVA) and multiple logistic regression analysis. In this study, the prevalence of NAFLD or AFLD was used as a dependent variable, and the quartiles of C3 as independent variables. For characteristics analysis, differences among C3 categories were examined using ANOVA for continuous variables, and logistic regression analysis for proportional variables. Bonferroni corrected P values were used for comparisons between C3 quartiles. The relationship between C3 categories and prevalence of NAFLD or AFLD were examined by multiple logistic regression analysis, after adjustment for the following covariates: log-transformed age, sex, log-transformed BMI, smoking status, drinking status, log-transformed ALT, AST and GGT as well as family history of CVD, hypertension, hyperlipidemia, or diabetes. Odds ratio (OR) and a 95% CI were calculated. Because the number of AFLD in female (n = 7) was too small to perform multiple logistic regression analysis [20], females were excluded from final analysis. A linear trend across increasing quartiles was tested using the median value of each quartile as an ordinal variable. The interaction was assessed by testing the interaction term added to the adjusted model as a covariate. All tests were two tailed and *P*<0.05 was defined as statistically significant.

## Results

In this study, 60.8% (1,779 of 2,926) of participants were male and 39.2% (1,147 of 2,926) were female, with mean ages (SD) of 50.2 (10.2) and 50.1 (10.6) years, respectively.

The prevalence of NAFLD and AFLD in men and women were 37.3% (1,020 of 2,733) and 10.1% (193 of 1,906), respectively. Characteristics of participants across quartiles of C3 are presented in Table 1. Compared with participants in the lowest quartile of C3, participants in the upper three quartiles tended to be older, have higher BMI, waist circumferences, as well as levels of TC, TG, LDL, ALT, AST and GGT, a higher FBS and BP (systolic and diastolic), but lower HDL; in addition, a higher proportion of these participants were male and a lower proportion of nonsmokers. Other than these results, no significant differences were observed between participants in different C3 quartiles. Similar results were observed in the AFLD study population, excluding a significant difference in the number of current smokers (Table 2).

**Table 1:** Participant characteristics by quartiles of complement C3 (n = 2,733) ^a^.

	Quartiles of complement C3 (g/L, range)				
	Level 1 (0.53–0.89)	Level 2 (0.90–1.02)	Level 3 (1.03–1.13)	Level 4 (1.14–1.93)	*p* for trend ^b^
	(n = 689)	(n = 734)	(n = 590)	(n = 720)	
Age (y)	47.6 (46.8, 48.3) ^c^	49.1 (48.3, 49.8)	50.6 (49.7, 51.4)	49.2 (48.4, 49.9)	<0.0001
Sex (males, %)	48.9	62.4	61.9	60.7	<0.0001
BMI (kg/m2)	23.4 (23.2, 23.6)	25.1 (24.8, 25.3)	25.7 (25.5, 26)	27.1 (26.9, 27.4)	<0.0001
Waist circumference (cm)	79.0 (78.3, 79.7)	84.9 (84.2, 85.7)	86.7 (85.9, 87.5)	90.6 (89.9, 91.4)	<0.0001
TC (mmol/L)	4.63 (4.57, 4.7)	4.85 (4.79, 4.92)	4.97 (4.9, 5.05)	5.20 (5.13, 5.27)	<0.0001
TG (mmol/L)	0.98 (0.95, 1.02)	1.34 (1.29, 1.39)	1.46 (1.40, 1.52)	1.77 (1.71, 1.84)	<0.0001
LDL (mmol/L)	2.54 (2.48, 2.59)	2.80 (2.74, 2.86)	2.90 (2.83, 2.97)	3.05 (2.98, 3.11)	<0.0001
HDL (mmol/L)	1.52 (1.50, 1.55)	1.32 (1.30, 1.35)	1.29 (1.27, 1.32)	1.24 (1.22, 1.26)	<0.0001
SBP (mmHg)	118.4 (117.2, 119.6)	122.5 (121.4, 123.7)	126.2 (124.9, 127.6)	128.8 (127.5, 130.1)	<0.0001
DBP (mmHg)	74.5 (73.7, 75.3)	77.5 (76.7, 78.3)	79.7 (78.7, 80.6)	82.2 (81.3, 83.1)	<0.0001
FBS (mmol/L)	5.04 (4.98, 5.10)	5.25 (5.19, 5.31)	5.39 (5.32, 5.46)	5.52 (5.46, 5.58)	<0.0001
ALT(mmol/L)	14.2 (13.7, 14.8)	17.8 (17.2, 18.5)	20 (19.2, 20.9)	24.3 (23.4, 25.2)	<0.0001
AST(mmol/L)	17.1 (16.7, 17.5)	18.2 (17.8, 18.6)	19.0 (18.6, 19.5)	20.7 (20.2, 21.2)	<0.0001
GGT(mmol/L)	17.5 (16.7, 18.4)	22.9 (21.9, 24.0)	26.1 (24.8, 27.4)	32.4 (31.0, 33.9)	<0.0001
Smoking status (%)					
Smoker	23.1	31.3	30.3	28.1	0.09
Ex-smoker	0.15	0.00	0.00	0.00	0.92
Nonsmoker	76.5	68.7	69.5	71.8	0.04
Drinker (%)	40.4	43.6	44.9	44.9	0.08
Family history of diseases (%)					
CVD	28.3	32.6	30.2	30.1	0.68
Hypertension	40.6	39.5	42.4	41.2	0.59
Hyperlipidemia	0.00	0.14	0.00	0.00	0.66
Diabetes	18.3	16.4	21.0	20.6	0.09

^a^ BMI, body mass index; TC, total cholesterol; TG, triglycerides; LDL, low density lipoprotein cholesterol; HDL, high-density lipoprotein-cholesterol; SBP, systolic blood pressure; DBP, diastolic blood pressure; FBS, fasting blood sugar; ALT, alanine aminotransferase; AST, aspartate aminotransferase; GGT, gamma-glutamyl transpeptidase; CVD, cardiovascular disease.

^b^ Analysis of variance or logistic regression analysis.

^c^ Geometric mean (95% confidence interval) (all such values).

**Table 2:** Participant characteristics by quartiles of complement 3 in males (n = 978) ^a^.

	Quartiles of complement 3 (g/L, range)				
	Level 1 (0.54–0.88)	Level 2 (0.88–0.98)	Level 3 (0.99–1.09)	Level 4 (1.11–1.93)	*p* for trend ^b^
	(n = 245)	(n = 246)	(n = 234)	(n = 253)	
Age (y)	50.1 (48.8, 51.5) ^c^	50.2 (48.9, 51.6)	49.0 (47.7, 50.4)	46.9 (45.7, 48.2)	0.25
BMI (kg/m2)	24.0 (23.7, 24.4)	24.9 (24.5, 25.2)	25.6 (25.2, 25.9)	26.6 (26.2, 26.9)	<0.0001
Waist circumference (cm)	84.4 (83.4, 85.4)	87.4 (86.4, 88.4)	89.4 (88.3, 90.4)	92.3 (91.2, 93.4)	<0.0001
TC (mmol/L)	4.60 (4.50, 4.70)	4.87 (4.76, 4.98)	4.92 (4.81, 5.03)	5.12 (5.01, 5.23)	<0.0001
TG (mmol/L)	1.12 (1.05, 1.19)	1.35 (1.27, 1.44)	1.51 (1.41, 1.61)	1.72 (1.62, 1.83)	<0.0001
LDL (mmol/L)	2.59 (2.50, 2.68)	2.81 (2.72, 2.91)	2.87 (2.77, 2.98)	3.01 (2.91, 3.12)	<0.0001
HDL (mmol/L)	1.39 (1.35, 1.44)	1.32 (1.28, 1.36)	1.25 (1.22, 1.29)	1.24 (1.21, 1.28)	<0.0001
SBP (mmHg)	122.1 (120.1, 124.1)	124.6 (122.6, 126.7)	126.1 (124, 128.2)	126.8 (124.8, 128.9)	<0.01
DBP (mmHg)	78.1 (76.7, 79.5)	79.8 (78.4, 81.3)	80.8 (79.4, 82.4)	83.2 (81.7, 84.6)	<0.01
FBS (mmol/L)	5.14 (5.04, 5.24)	5.28 (5.18, 5.39)	5.33 (5.23, 5.44)	5.44 (5.34, 5.55)	<0.01
ALT(mmol/L)	15.8 (14.9, 16.7)	18.7 (17.6, 19.8)	20.4 (19.2, 21.7)	24.3 (23.0, 25.8)	<0.0001
AST(mmol/L)	17.3 (16.6, 17.9)	18.5 (17.8, 19.3)	18.7 (18.0, 19.5)	20.4 (19.6, 21.2)	<0.01
GGT(mmol/L)	23.5 (21.8, 25.4)	27.6 (25.6, 29.8)	30.7 (28.4, 33.2)	40.3 (37.4, 43.4)	<0.0001
Smoking status (%)					
Current smoker	47.8	48.8	46.2	44.7	0.40
Ex-smoker	0.41	0.00	0.00	0.00	-
Nonsmoker	51.4	51.2	53.8	55.3	0.37
Drinker (%)	65.3	65.0	67.5	80.6	<0.001
Family history of diseases (%)					
CVD	24.1	34.2	29.5	31.2	0.19
Hypertension	38.4	41.5	39.3	43.5	0.34
Hyperlipidemia	0.00	0.00	0.00	0.00	-
Diabetes	15.9	13.0	17.1	19.8	0.14

^a^ BMI, body mass index; TC, total cholesterol; TG, triglycerides; LDL, low density lipoprotein cholesterol; HDL, high-density lipoprotein-cholesterol; SBP, systolic blood pressure; DBP, diastolic blood pressure; FBS, fasting blood sugar; ALT, alanine aminotransferase; AST, aspartate aminotransferase; GGT, gamma-glutamyl transpeptidase; CVD, cardiovascular disease.

^b^ Analysis of variance or logistic regression analysis.

^c^ Geometric mean (95% confidence interval) (all such values).

Table 3 shows the crude and adjusted association between quartiles of C3 and NAFLD. In the final multivariate models, the adjusted OR (95% CI) for NAFLD across C3 quartiles were 1.00 (reference), 2.25 (1.63, 3.12), 3.03 (2.19, 4.23) and 4.13 (2.97, 5.77) (*P* for trend<0.0001), respectively. Similar results were also observed when males and females were analyzed separately. In final mode, the adjusted ORs (95% CI) for NAFLD across the quartiles of C3 were as follows: for males, the values were 1.00, 1.78 (1.26, 2.51), 2.45 (1.72, 3.51) and 2.60 (1.79, 3.78) (P for trend<0.0001), respectively; for females, the values were 1.00, 3.08 (1.31, 8.51), 4.26 (1.86, 11.57) and 7.29 (3.23, 19.60) (P for trend<0.0001), respectively (interaction P value = 0.12).

**Table 3:** Adjusted odds ratios of quartiles of complement 3 to NAFLD ^a^.

	Quartiles of complement 3 (g/L, range)				
	Level 1 (0.53–0.89)	Level 2 (0.90–1.02)	Level 3 (1.03–1.13)	Level 4 (1.14–1.93)	*p* for trend ^b^
	(n = 689)	(n = 734)	(n = 590)	(n = 720)	
No. of NAFLD	80	244	261	435	-
Model 1 ^d^	1.00	3.79 (2.88, 5.04) ^c^	6.04 (4.57, 8.06)	11.6 (8.85, 15.4)	<0.0001
Model 3 ^e^	1.00	2.67 (1.96, 3.67)	4.06 (2.96, 5.61)	6.56 (4.80, 9.04)	<0.0001
Model 5 ^f^	1.00	2.25 (1.63, 3.12)	3.03 (2.19, 4.23)	4.13 (2.97, 5.77)	<0.0001

^a^ NAFLD, non-alcoholic fatty liver disease; BMI, body mass index.

^b^ Multiple logistic regression analysis.

^c^ Adjusted odds ratios (95% confidence interval) (all such values).

^d^ Crude model.

^e^ Adjusted for log-transformed age, sex and log-transformed BMI.

^f^ Additionally adjusted for smoking status, drinking status, and family history of cardiovascular disease, hypertension, diabetes and log-transformed ALT, AST and GGT.

Table 4 indicates the crude and adjusted associations between the quartiles of C3 and AFLD in males. After adjustment for potential confounders, the ORs (95% CI) of AFLD for increasing quartiles of C3 were 1.00, 1.72 (0.86, 3.53), 2.95 (1.54, 5.90) and 2.09 (1.08, 4.18) (*P* for trend = 0.02).

**Table 4:** Adjusted odds ratios of quartiles of complement 3 to AFLD in males (n = 978) ^a^.

	Quartiles of complement 3 (g/L, range)				
	Level 1 (0.54–0.88)	Level 2 (0.88–0.98)	Level 3 (0.99–1.09)	Level 4 (1.11–1.93)	*p* for trend ^b^
	(n = 245)	(n = 246)	(n = 234)	(n = 253)	
No. of AFLD	16	34	58	78	-
Model 1 ^d^	1.00	2.30 (1.25, 4.38) ^c^	4.72 (2.68, 8.74)	6.38 (3.69, 11.68)	<0.0001
Model 2 ^e^	1.00	1.88 (0.98, 3.72)	3.41 (1.86, 6.54)	3.10 (1.69, 5.94)	<0.0001
Model 3 ^f^	1.00	1.72 (0.86, 3.53)	2.95 (1.54, 5.90)	2.09 (1.08, 4.18)	0.02

^a^ AFLD, alcoholic fatty liver disease; BMI, body mass index.

^b^ Multiple logistic regression analysis.

^c^ Adjusted odds ratios (95% confidence interval) (all such values).

^d^ Crude model.

^e^ Adjusted for log-transformed age, sex and log-transformed BMI.

^f^ Additionally adjusted for smoking status, drinking status, and family history of cardiovascular disease, hypertension, diabetes and log-transformed ALT, AST and GGT.

## Discussion

In this cross-sectional study, we investigated the association between serum C3 levels and FLD in an adult population. We have demonstrated for the first time that serum C3 is independently associated with both NAFLD and AFLD, after adjustment for potential confounding factors.

The liver is the primary site of C3 synthesis [21]. However, it has been demonstrated that a number of other cells can also synthesize and secrete C3, such as activated macrophages at inflammation sites and adipose tissue [22, 23]. The present study assessed serum C3 concentration of, but not its origin. Thus, further study is needed to explore the origin of serum C3 and its value towards the prevention and treatment of FLD.

Our findings are in keeping with previous data reported by in adult populations of Turks and Netherlanders [12–14]. In a small case-control study examining 46 NAFLD Turks, C3 levels were significantly higher in NAFLD patients compared with healthy controls, and C3 activity correlated with disease severity [13]. Consistently, in group of 43 severely obese Netherlanders with NAFLD, who underwent liver biopsies, activated C3 deposition was abundant around steatotic hepatocytes [12]. Another cross-sectional study involving 523 middle-aged and older Dutch subjects selected on the basis of increased risk of metabolic and cardiovascular disease (CVD), found that an elevated C3a level was related to liver fat percentage [14]. The study also showed that C3a was related to liver enzymes score, a robust measure of hepatocellular injury, in heavy alcohol consumers. One caveat of that study was a lack of systematic diagnosis of FLD using reliable imaging techniques. C3a is a cleavage product of C3, but C3a levels do not always correlate precisely with C3 levels [23]. It is likely that general complement activation and C3-specific activity have shown distinct and different relationships with FLD [24]. Thus, in our study, we set out to and succeeded in demonstrating a direct correction between C3 levels (rather than the activated complement components) and Fatty Liver Disease in a large sample of Chinese adults.

Activation of the complement system has been implicated in the pathogenesis of FLD. The pathogenesis of NAFLD and AFLD may be different. Alcohol-induced hepatotoxicity and oxidative stress are important mechanisms contributing to alcoholic liver injury [25]. Excessive ethanol consumption induces an imbalance in lipid metabolism in the liver, resulting in increased lipogenesis, reduced lipolysis, reduced cyclic AMP-activated protein kinase activity, production of reactive oxygen species and pro-inflammatory cytokines, as well as activation of natural killer cells [9]. A previous study examining C3 deficiency showed that C3 is an important regulator of lipid metabolism [26]. In addition, several animal studies attempted to elucidate the precise role of complement in the pathogenesis of AFLD. In the AFLD animal model, activated the complement components were deposited in the liver [27, 28], and C3-deficient animals tend to reduce fatty infiltration and steatosis after ethanol-induced liver damage [29, 30], implying that a low level of C3 is beneficial to fatty deposits in liver. On the other hand, the innate immune response in the liver plays an important role during the progression of NAFLD. Saturated fatty acids represent an endogenous danger in the form of a first hit (hepatic steatosis), which can up-regulate the inflammasome. Hepatocytes exposed to saturated fatty acids release danger signals that trigger inflammasome activation in immune cells [31]. Alcohol and fatty acid may stimulate the immune system in different ways to increase serum C3 levels, and induce the progression of FLD.

Several studies have shown that C3 is a risk factor for incident diabetes as well as cardiometabolic disorder [14–16]. The current body of evidence also strongly suggests that NAFLD was associated with increased risk of CVD [17]. Therefore, we postulate that C3 might increase the risk of CVD due to NAFLD. Further study is needed to explore this hypothesis.

There are several limitations to our study. First, liver biopsy, the gold standard in the diagnosis of liver disease, was not available in present study, due to the apparently healthy study population. Instead, we used hepatic ultrasonography scanning to detect fatty liver. This technique has a sensitivity of 89% and a specificity of 93% [32] and is wildly used in population-based studies due to its noninvasiveness and easy accessibility [33]. Second, the prevalence of NAFLD is relatively high compared to other studies using Chinese adults [8]. This may be related to the fact that the middle-aged and elderly subjects who participated in our study have a higher risk for metabolic disease and FLD. Third, we measured serum C3 levels, but not levels of its activated components, such as C3a or C3b. While C3a may act as a pro-inflammatory agent, several studies have shown that C3 (rather than C3a) is an important predictive factor for metabolic syndrome, insulin resistance and hypertension [15, 34–36]. Thus, C3 maybe a more useful predictor for FLD than its activated components. Other limitations include the cross-sectional design of this study, which does not allow us to draw definite conclusions regarding potential causal relationships; further prospective studies should be carried out in order to establish whether or not a causal relationship exists between C3 levels and FLD.

In conclusion, this study is the first to show that serum C3 level is independently associated to NAFLD and AFLD in an adult population. Further studies are needed to explore the potential causality and precise role of C3 in FLD pathogenesis.
